# A Leaky-Integrator Model as a Control Mechanism Underlying Flexible Decision Making during Task Switching

**DOI:** 10.1371/journal.pone.0059670

**Published:** 2013-03-22

**Authors:** Akinori Mitani, Ryo Sasaki, Masafumi Oizumi, Takanori Uka

**Affiliations:** 1 Department of Neurophysiology, Graduate School of Medicine, Juntendo University, Bunkyo, Tokyo, Japan; 2 Faculty of Medicine, The University of Tokyo, Bunkyo, Tokyo, Japan; 3 Neuroscience Graduate Program, University of California San Diego, La Jolla, California, United States of America; 4 Department of Brain and Cognitive Sciences, Center for Visual Science, University of Rochester, Rochester, New York, United States of America; 5 Laboratory for Mathematical Neuroscience, RIKEN Brain Science Institute, Wako, Saitama, Japan; 6 Department of Psychiatry, University of Wisconsin, Madison, Wisconsin, United States of America; National Research & Technology Council, Argentina

## Abstract

The ability to switch between tasks is critical for animals to behave according to context. Although the association between the prefrontal cortex and task switching has been well documented, the ultimate modulation of sensory–motor associations has yet to be determined. Here, we modeled the results of a previous study showing that task switching can be accomplished by communication from distinct populations of sensory neurons. We proposed a leaky-integrator model where relevant and irrelevant information were stored separately in two integrators and task switching was achieved by leaking information from the irrelevant integrator. The model successfully explained both the behavioral and neuronal data. Additionally, the leaky-integrator model showed better performance than an alternative model, where irrelevant information was discarded by decreasing the weight on irrelevant information, when animals initially failed to commit to a task. Overall, we propose that flexible switching is, in part, achieved by actively controlling the amount of leak of relevant and irrelevant information.

## Introduction

A crucial aspect of human cognitive flexibility is our ability to respond differently to identical sensory inputs depending on the task. The process of encoding task information is well studied; recent physiological studies have shown that neurons encode abstract rules and have demonstrated task-related neuronal modulation in the prefrontal cortex and other regions [1]–[4]. Meanwhile, task-dependent modulation of sensory–motor associations determines how task information ultimately controls our behavior. Despite its being an essential part of our flexibility, this process is still poorly understood.

To address this issue, Sasaki and Uka applied task switching to a perceptual decision-making task [5]. Based on extensive documentation that middle temporal (MT) neurons contribute to both direction [6]–[8] and depth [9]–[11] discrimination, MT neuron responses were measured while monkeys switched between the two tasks. Sasaki and Uka found that although sensitivities of MT neurons were task independent, some of the neurons whose preferred direction and preferred depth were related to opposite choices in the two tasks (incongruent neurons) showed covariation with behavioral choices in either of the two tasks [5]. They suggested that sensory neurons are divided into distinct populations for each task and that task switching is accomplished by attending to information from task-relevant neurons and discarding information from task-irrelevant neurons.

We examined two possible mechanisms of this selective readout. In both mechanisms, we considered evidence-accumulation models where reliable decisions are generated by integrating responses of sensory neurons [12]. We did not explicitly implement biologically realistic networks of the integrators to build a thorough mechanistic model, but here we aimed at establishing a phenomenological model for better understanding of computational mechanisms of the readout. One mechanism for discarding the task-irrelevant information, which one would simply imagine, is to decrease its weight. The other one, which seems like a rather indirect way, is to increase the leakage of the integrator. We call the former mechanism a gated-integrator model and the later one a leaky-integrator model.

We compared these models by simulations based on the recorded activities of MT neurons [5]. In the gated-integrator model, the weight on the task-irrelevant information needed to be small at first and then gradually increase in order to explain the time course of the covariation between behavior choices and neural data. The leaky-integrator model equally explained the time course as the gated-integrator model under the assumption that task-relevant information was stored in a perfect integrator and that task-irrelevant information was stored in a leaky integrator. These models were equivalent under fixed evidence accumulation duration. When the duration was shortened, the performance of the leaky-integrator model deteriorated as one can naturally expect [13], but the performance of the gated-integrator model counterintuitively improved. In addition, when task commitment was delayed, the performance of the leaky-integrator model did not deteriorate as rapidly as the gated-integrator model because stored task-irrelevant information could be discarded later through leakage. From these results, we propose the leaky-integrator model as a potential mechanism for flexible task switching.

## Materials and Methods

### General

The psychophysical and electrophysiological data were acquired from a previous study [5]. Because detailed experimental procedures were described previously, here we explain the outline of the experiment and focus on data analysis and simulations. Two Japanese monkeys (*Macaca fuscata*) were used in this study. Animal care and experimental procedures were approved by the Juntendo University Animal Care and Use Committee and were in accordance with NIH guidelines.

### Experimental Procedures

The monkeys were seated in a monkey chair in front of a color CRT with the head in a fixed position. The positions of both eyes were monitored and stored at 200 Hz using the eye-coil technique [14], [15]. Stereoscopic images were displayed by presenting the left and right half images alternately at 100 Hz (50 Hz for each eye). The monkeys viewed the display through a pair of ferroelectric shutters synchronized to the video refresh.

**Figure 1:**
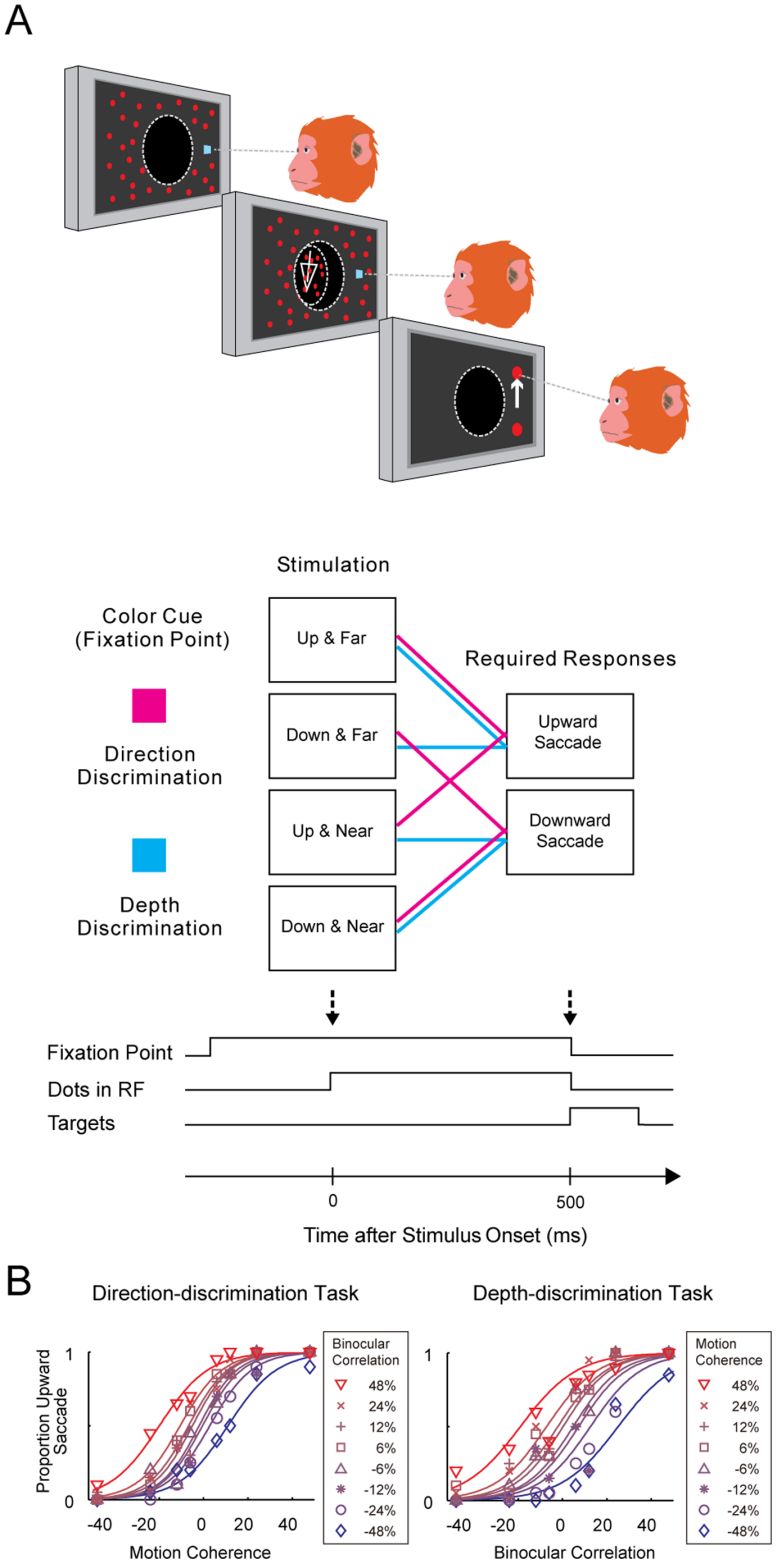
Task and animal performance. Adapted from [5]. (A) Sequence of events. After the monkeys fixated, the stimulus was presented on the screen for 500 ms. Two choice targets then appeared above and below the fixation point. During each trial, the color of the fixation point (magenta or cyan) indicated whether the monkey was to discriminate direction (UP or DOWN) or depth (FAR or NEAR) using saccadic eye movements (upward or downward, respectively). RF, receptive field. (B) An example psychometric function from a single session.

Monkeys were trained to switch between a direction-discrimination task and a depth-discrimination task while isolated neurons were recorded from the MT area (Fig. 1A). For each trial, the color of the fixation point (magenta or cyan) indicated whether the monkeys should discriminate direction or depth, respectively. The tasks were randomly interleaved from trial to trial. After the monkeys fixated for 300 ms, a random-dot stereogram (RDS) moving in one of two directions at one of two binocular disparities appeared in the receptive field of the recorded neuron for 500 ms. In the direction-discrimination task, the monkeys indicated whether the coherently moving dots moved up or down; in the depth-discrimination task, the monkeys indicated whether the correlated dots were farther or nearer than the plane of fixation by making a saccade to one of two targets appearing above and below the fixation point immediately after the offset of the RDS. The fixation point and the RDS were turned off when the two saccade targets appeared, and the monkeys were required to make a saccade within 1 s after the appearance of the two saccade targets. Correct responses were rewarded with a drop of water or juice. Task difficulty was titrated by changing the percentage of coherently moving dots (motion coherence) or binocularly correlated dots (binocular correlation) in the RDS. Motion coherence and binocular correlation were varied independently from trial to trial.

Single-unit recordings were made in the MT area using tungsten microelectrodes (impedance, 0.2–2 MΩ at 1 kHz) that were advanced into the cortex through a transdural guide tube. Spike times and behavioral event markers were stored to disk with 1-ms resolution.

### Data Analysis and Simulation

All statistical analyses and simulation were performed using MATLAB software (MathWorks, Natick, MA, USA).

#### Behavioral analysis

We analyzed the behavioral choice data following Sasaki and Uka [5] with logistic regression; the probability of an upward saccade (*p_up_*) was given by the following equations:
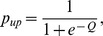


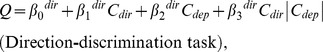


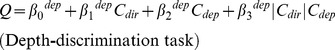

*C_dir_* denote motion coherence and and *C_dep_* denote binocular correlation. Positive values of *C_dir_* and *C_dep_* indicate coherence/correlation with upward motion or far depth, whereas negative values indicate coherence/correlation with downward motion or near depth. *β_0_* accounts for offset, and *β_1_* and *β_2_* account for sensitivity to motion coherence and binocular correlation. We included the interaction term, *β_3_*, assuming that a strong irrelevant stimulus may degrade sensitivity.

To determine whether monkeys performed the wrong task stochastically, we also tested a nine-parameter model assuming that the monkeys performed the wrong task with a probability of *p_err_*:
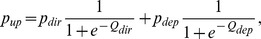


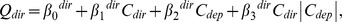


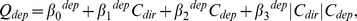


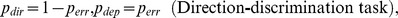


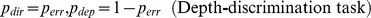



This nine-parameter model was compared with the previous eight-parameter model by calculating Akaike’s Information Criteria (AIC) and estimating *p_err_*.

To quantify how well the monkeys switched between direction discrimination and depth discrimination, we used the average switch ratio (SR) calculated from the sensitivities of direction or depth in the relevant and irrelevant tasks:
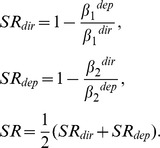



#### Choice probability

We calculated choice probability (CP) to elucidate the relationship between MT activity and behavioral choice [8]. To quantify the difference between the two spike-count distributions associated with the neuron’s preferred and null choices, we used receiver-operating-characteristic (ROC) analysis and measured the area under the ROC curve, which is termed CP. To calculate the CP time course, the responses during each trial were divided into eight 100-ms bins (starting −100 ms, 0 ms, 100 ms, 200 ms, 300 ms, 400 ms, 500 ms and 600 ms after stimulus onset). The responses in each bin were *z*-scored for each motion coherence/binocular correlation condition and sorted by choice (preferred vs. null). *Z*-scored responses were then combined separately across neurons that preferred direction and depth related to the same choice (congruent neurons) and neurons that preferred direction and depth related to opposite choices (incongruent neurons); the CPs were calculated from these distributions. Confidence intervals were approximated using the 2.5th percentile and 97.5th percentile of the distribution of 10000 bootstraps calculated by independent resampling with replacement from the *z*-scored distributions [16]. Conditions under which monkeys made upward choices fewer than 1/4 or more than 3/4 of the trials were excluded.

#### Gated-integrator and leaky-integrator models

We examined a gated-integrator model, a time-varying-gate model and two leaky-integrator models to investigate the mechanism of task switching. All models consisted of three steps: 1) sensory representation by MT neurons; 2) temporal integration of MT responses; and 3) decision making based on the integrated variables.

Simulated MT responses for each stimulus condition were generated based on the average responses of the whole population of recorded MT neurons [5]. First, each recorded neuron’s spike rate for each stimulus condition was estimated using the kernel-density estimate [17]. The recorded spike trains were convolved with a Gaussian kernel with a standard deviation of 20 ms, defined from −60 ms to 60 ms. The bandwidth of 20 ms was selected using kernel bandwidth optimization [17] to create a smooth spike-rate function without blunting the rising point. Spike rates were calculated numerically with a step size of 1 ms. The spike rates were then averaged across all neurons (*N* = 117) for each motion coherence/binocular correlation condition separately for the two tasks to generate “typical responses” of MT neurons. Averaged spike rates (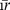) were calculated with the following equation:
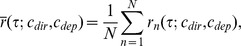
where *r_n_* is the estimated spike rate of a neuron *n* ( = 1, 2,…, 117) at time *τ* after stimulus onset with motion coherence *c_dir_* and binocular correlation *c_dep_*. Here, positive values of *c_dir_* and *c_dep_* indicate coherence/correlation at the neuron’s preferred direction or depth, whereas negative values indicate coherence/correlation at the neuron’s null direction or depth.

Both the gated-integrator model and the leaky-integrator model consisted of eight simulated MT neurons (Table 1). Simulated trials were numbered by *t* for each coherence (*C_dir_*)/correlation (*C_dep_*) combination. Positive values of *C_dir_* indicate coherence for upward stimuli and *C_dep_* indicate correlation for far stimuli. The responses of the simulated neurons to a particular stimulus on a given trial, *s_n_*(*τ*; *C_dir_*, *C_dep_*, *t*) (*n* = 1, 2,…, 8), were generated as a function of time, *τ*, for each 1-ms bin by Poisson spike generators. The averaged spike rates for the eight simulated neurons complied to (*c_dir_, c_dep_*), where (*c_dir_*, *c_dep_*) = (*C_dir_*, *C_dep_*), (−*C_dir_*, *C_dep_*), (*C_dir_*, −*C_dep_*), (−*C_dir_*, −*C_dep_*) depending on the preference of the neuron shown in Table 1. Hereafter, the variable *τ* and the parameters *C_dir_*, *C_dep_* and *t* will be omitted when obvious. *s_n_* was 1 spike/ms in a bin with a spike and 0 spike/ms in a bin without a spike. Simulated instantaneous differential responses for direction (up/down: *i_dir_*) and depth (far/near: *i_dep_*) were calculated using the following equations:

**Table 1:** Properties of eight simulated neurons.

Neuron	Preferred Direction	Preferred Depth	Congruent/Incongruent	Contribution to Task
1	Up	Far	Congruent	Both
2	Up	Far	Congruent	Both
3	Down	Far	Incongruent	Depth
4	Down	Far	Incongruent	Direction
5	Up	Near	Incongruent	Depth
6	Up	Near	Incongruent	Direction
7	Down	Near	Congruent	Both
8	Down	Near	Congruent	Both

Here, responses to upward motion and far depth were described using positive values.

**Figure 2:**
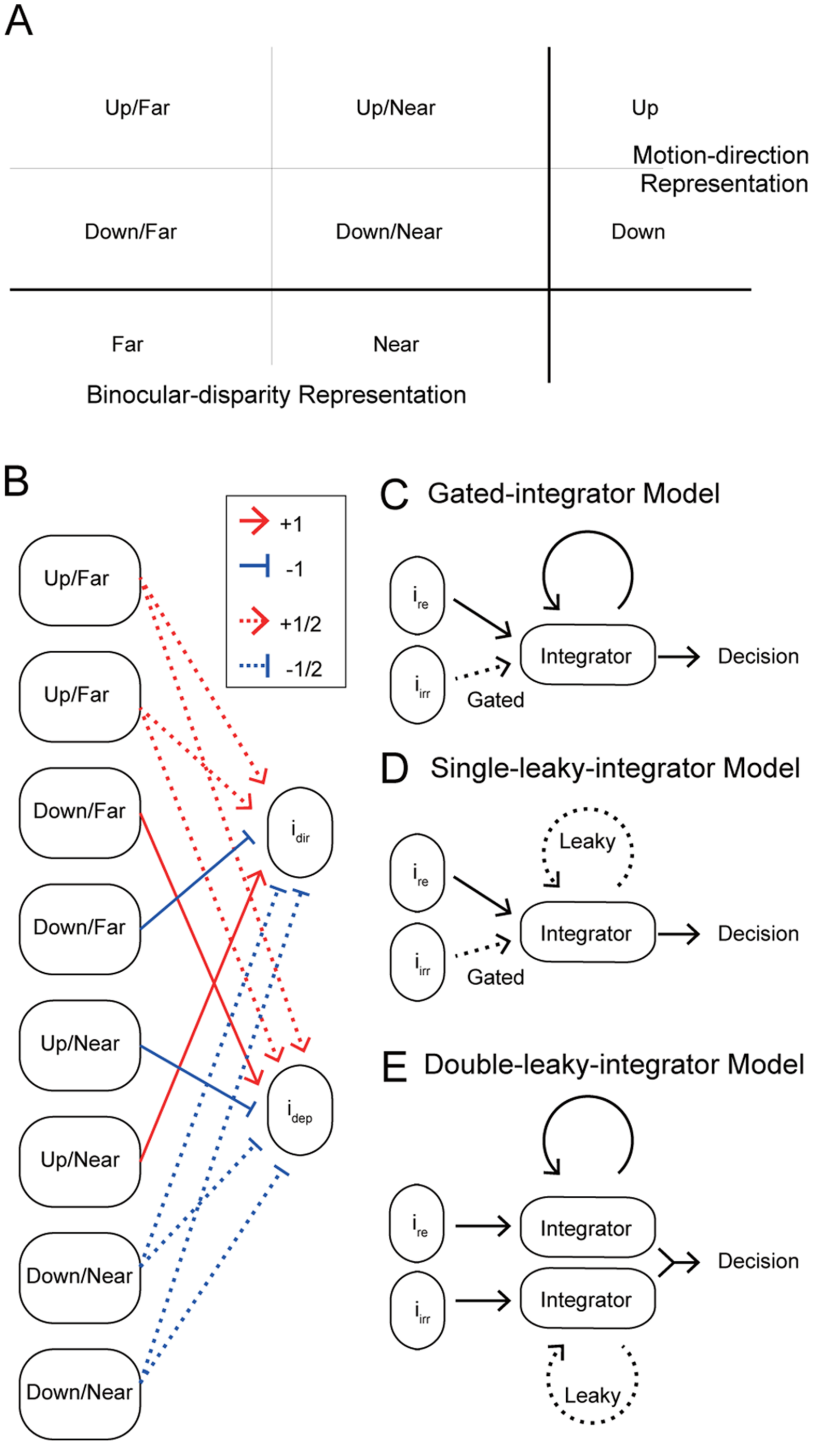
Schematic illustration of the models. (A) Readout of direction or depth from the responses of middle temporal (MT) neurons. The difference between an average response of up/far and up/near neurons and an average response of down/far and down/near neurons represented motion direction signals. The difference between an average response of up/far and down/far neurons and an average response of up/near and down/near neurons represented binocular disparity signals. (B) A schematic diagram of how MT neurons were connected to the integrators in the model. (C) Gated-integrator model, (D) single-leaky-integrator model and (E) double-gated-integrator model



Next, simulated instantaneous differential responses were integrated. Here, *i_dir_* and *i_dep_* were assigned to be either relevant or irrelevant depending on the task. For example, *i_rrelevant_* = *i_dir_* and *i_irrelevant_* = *i_dep_* in the direction-discrimination task and vice versa in the depth-discrimination task. The four models, the gated-integrator model (Fig. 2C), the time-varying-gate model, the single-leaky-integrator model (Fig. 2D), and the double-leaky-integrator model (Fig. 2E) can be regarded as variations of a more general model. In this general model, the integrated value *I* at time *T* was given by the following equations:

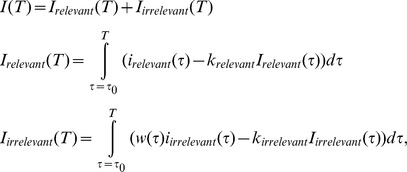
where *τ*_0_ is 100 ms after stimulus onset, the time when stimulus-induced responses arise in MT neurons and *τ*_1_ is 100 ms after stimulus offset; *k_relevant_* and *k_irrelevant_* determines the rate of leakage, and *w*(*τ*) is the weight on the irrelevant information. When *k_relevant_* and *k_irrelevant_* are identical, the equations can be reduced to one integration by assuming *k* = *k_relevant_* = *k_irrelevant_* (the gated-integrator model, the time-varying-gate model and the single-leaky-integrator model);



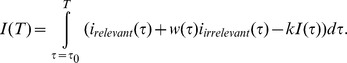



The gated-integrator model was defined by setting *k_relevant_* = *k_irrelevant_* = 0 and *w*(*τ*) = *w* (constant). The time-varying-gate model was defined by setting *k_relevant_* = *k_irrelevant_* = 0 and *w*(*τ*) = exp(*k*(*τ*−*τ*_1_)). We assumed an exponentially increasing weight to compare with the leaky-integrator model below. The single-leaky-integrator model was defined by setting *k_relevant_* = *k_irrelevant_* = *k* and *w*(*τ*) = *w* (constant). The double-leaky-integrator model was defined by setting *k_relevant_* = 0, *k_irrelevant_* = *k* and *w*(*τ*) = 1.

The integration was calculated in 1-ms steps. After 500 ms of integration, the integrated value was evaluated. If the value was positive, an upward saccade was selected, and if the value was negative, a downward saccade was selected. This calculation was conducted at each coherence/correlation combination for 1000 trials for all simulations.

To match the sensitivities between behavioral and model, we selected a population of neurons with roughly equal sensitivity for direction and depth on average. Neuronal sensitivity was quantified by neurometric thresholds which were computed using a receiver operating characteristic analysis as previously described [5]. Neurons were sorted by the ratio between direction and depth thresholds, and 40 neurons were selected so that the sensitivity for direction and depth were equal on average.

## Results

### Description of Previous Results

In this section, we briefly describe the results obtained by Sasaki and Uka [5]. Sasaki and Uka applied task switching to a perceptual decision paradigm to study how task switching modulates sensory–motor associations. Two monkeys were trained to discriminate either the direction or depth of RDS. During the direction-discrimination task, stimuli with an upward-motion component were associated with upward saccades, and those with a downward-motion component were associated with downward saccades. During the depth-discrimination task, stimuli having far disparity were associated with upward saccades, and those having near disparity were associated with downward saccades (Fig. 1A).

Examples of psychometric functions from one recording session are shown in Figure 1B. The left panel shows the proportion of upward saccades in a direction-discrimination task as a function of motion coherence for each binocular correlation. Although the monkey correctly discriminated between the two opposite motion directions, the choices were biased so that the sigmoid psychometric functions were shifted horizontally depending on the strength of the binocular correlation. The right panel confirms the same points for the depth-discrimination task. To quantify how well the monkeys switched between direction discrimination and depth discrimination, SR was calculated from the sensitivities to direction or depth in the relevant and irrelevant tasks (see Materials and Methods). If the monkeys could switch perfectly, SR would be 1; if the monkeys were completely oblivious to the task demands, SR would be 0. The average SR for all the recorded sessions was 0.70, indicating that the monkeys correctly switched between the tasks, although not perfectly (see Fig. 4C in [5]). Decisions were based on both the relevant and irrelevant features of the stimulus, but the influence of the irrelevant feature was smaller than that of the relevant feature.

The choice bias was not mainly caused by task misapplication. To determine the amount of task misapplication, we analyzed the data shown in Fig. 1B with a model assuming that the monkeys performed the wrong task with a probability of *p_err_* (see Materials and Methods)_._ Considering the nonlinearity of the logistic function, *p_err_* was not a redundant parameter. However, the fit of the model was comparable with or without *p_err_*: the estimated *p_err_* was 0.047, and AIC was 425.4 with *p_err_* compared to 427.7 without *p_err_*. Therefore, although the monkeys may have occasionally applied the wrong task, the choice bias was mainly caused by interference, not by task misapplication. This result supports using SR to measure the amount of interference.

MT neuron responses did not show task dependency (see Fig. 3 in [5]). Therefore, task switching is not implemented before MT, and task may modulate readout from the MT. To assess the functional coupling between MT responses and perceptual decision, CP, the trial-to-trial covariation in MT responses and behavioral choices, were computed (see Materials and Methods). CP is 0.5 when the responses do not covary with behavioral choices. CP is 1 when the responses completely covary with behavioral choices. MT neurons were classified into congruent neurons and incongruent neurons according to their preferred direction and depth. Congruent neurons preferred up/far or down/near stimuli, which were associated with the same behavioral responses in the two tasks. Incongruent neurons preferred up/near or down/far stimuli, which were associated with different behavioral responses in the two tasks. CPs of congruent neurons were similarly larger than 0.5 for both tasks. In contrast, when the CP of an incongruent neuron was larger than 0.5 for one task, the CP for the other task was close to 0.5 (see Fig. 7A in [5]). From these results, Sasaki and Uka [5] concluded that task switching may be accomplished by reading out different populations of incongruent neurons.

**Figure 3:**
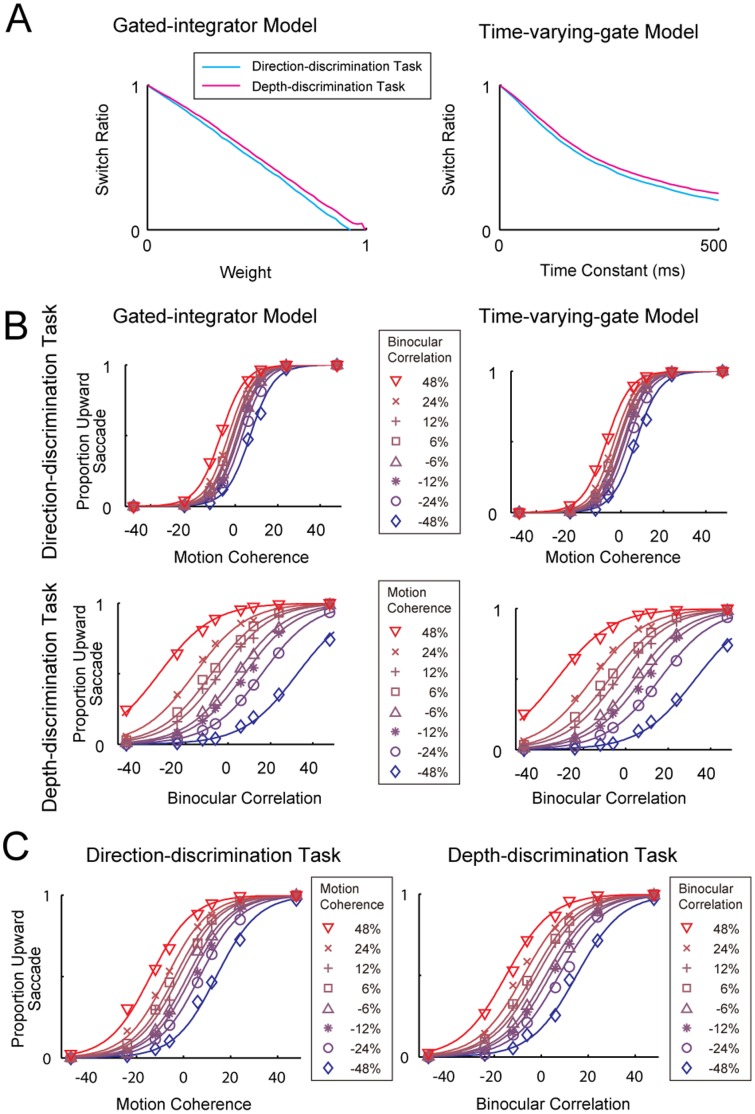
Neurometric function of the gated-integrator model and the time-varying-gate model. (A) Switch ratio (SR) decreased when the weight of the gated integrator increased or when the time constant of the time-varying gate lengthened. The parameters were determined to match the average behavioral SR of the two tasks. (B) Neurometric functions of the models using the parameters determined to match behavioral SR. (C) Neurometric function of the gated-integrator model based on the average firing rate of a selected group of neurons. Thresholds are 10.7% and 9.8%.

### A Gated-integrator Model can Explain the Behavioral Bias Observed during Task Switching

Using the responses of MT neurons described in Sasaki and Uka [5], we modeled these results in a quantitative manner. Our goal was to establish a simple phenomenological model of computation that explains both the behavioral and neuronal data. Although the model only contains a limited number of neurons, we do not imply that the brain only uses small numbers of neurons. Moreover, the connections in the models do not imply specific circuitry in the brain (See discussion). Rather, the neurons in the model can be thought of as representing a collective activity of a neuron group. We first investigated how dynamic readout of MT neurons may result in behavioral task switching. For simplicity, MT neurons were classified into four types depending on their preferences, up/far, down/far, up/near, and down/near, because most MT neurons have tuning to both direction and depth [18]. Information from one neuron is insufficient to determine the direction or depth of the stimulus. Rather, information from a population of neurons must be combined. For example, an average response of up/far and down/far neurons can indicate how far the stimulus is without being influenced by stimulus direction. In contrast, an average response of up/near and down/near neurons represents how near the stimulus is. In a popular model of decision making, these responses are accumulated and compete with one another to achieve a decision (race model; [19]). Alternatively, the diffusion model is based on the difference in these responses [20], [21]. The diffusion model has two integrators for two choices, and because of mutual inhibition, the differential responses are accumulated in effect. Several studies have implemented the diffusion model with parameters optimized to performance [22]–[24]. Additionally, the activities of ‘buildup’ neurons in the superior colliculus during two-choice decision tasks were better predicted by the diffusion model [25]. Therefore, here we employed the diffusion model and assumed that the difference between far and near responses underlies representation of stimulus binocular disparity by MT neurons. In the same way, the motion direction of the stimulus is represented by the difference between an average response of up/far and up/near neurons and an average response of down/far and down/near neurons (Fig. 2A).

A simple model of task switching is to switch the sign of the weight of incongruent neurons depending on the task while keeping the weight of congruent neurons constant. In this case, both congruent and incongruent neurons should have a CP>0.5 for both tasks. However, Sasaki and Uka [5] argued that incongruent neurons have a CP>0.5 in one of the two tasks, and that the CP of the other task is close to 0.5. Thus, they concluded that half the incongruent neurons were selectively read out in either task. Following this hypothesis, we represented each of the four groups of MT neurons with two simulated neurons, and each incongruent neuron contributed to one of the two tasks (Table 1, Fig. 2B). Congruent neurons were connected to both the direction and depth integrators. These connections do not necessarily indicate that there are direct physical connections between these neurons, but rather represent either direct or indirect contributions of these neurons to behavioral choices through representations of either direction or depth. Instantaneous direction and depth responses were represented as the difference in firing between a group of two congruent and one incongruent neurons and another group of two congruent and one incongruent neurons (see Materials and Methods). The responses of congruent neurons were halved to ensure balance with responses of incongruent neurons.

Simulations were composed of three steps, 1) sensory representation of the visual stimulus by MT neurons, 2) temporal integration of MT responses, and 3) decision making based on the integrated values. Responses of the eight simulated MT neurons (Table 1) were generated by a Poisson spike generator with the average spike rates of all recorded neurons (*N* = 117). The responses of these neurons were accumulated in an integrator. Integration started 100 ms after stimulus onset, when the stimulus-induced responses arose in the MT. The integration continued for 500 ms, the same length as the stimulus presentation. When the integration was over, a decision was made based on the sign of the sum of the integrators.

In theory, if the weights of the task-irrelevant inputs to the integrator decreased depending on task, this may explain incomplete task switching, observed in the monkeys’ behavior. The relevant feature-related responses mainly account for behavioral choice and the irrelevant feature-related responses are partially discounted. This gated-integrator model had one parameter to be determined; the weight of the gated irrelevant inputs. This parameter determined how well the model switched between the two tasks. We used SR to quantify switching performance. We determined the weight that matched the average behavioral SR of 0.70 (see Fig. 4C in [5]). Figure 3A (left) shows the SR of the model plotted as a function of weight. The weight varied from 0 to 1 in steps of 0.02, and the weight was determined to be 0.30. With this parameter, the gated-integrator model succeeded in explaining incomplete switching by the monkeys (Fig. 3B, left). However, the simulated sensitivities of the two tasks were different, even though there was little difference in the behavioral sensitivities (see Fig. 4B in [5]). This is because most MT neurons, and therefore the simulated neurons, were more sensitive to the motion than to the depth of the stimulus.

To roughly match thresholds between behavior and model, we hypothesized that neurons can be selectively read out. A population of neurons was selected so that this group of neurons had similar sensitivity for depth and direction on average. Based on the average firing rates of these neurons, we simulated the model and confirmed that the thresholds of the model matched the behavioral thresholds of the two monkeys (Fig. 3C). Behavioral thresholds of one monkey were 11.5% in the direction-discrimination task and 12.1% in the depth-discrimination task: for the other monkey, they were 14.0% and 12.7%. Thresholds were 10.7% and 9.8% for the model based on the average firing rates of the selected group of neurons. Thus, behavioral and model sensitivity can be matched. There are also other ways that behavioral and model sensitivity can be matched (see Discussion). In the following analysis, however, we used the original model where all recorded neurons were averaged since this is the simplest assumption, and it is not the central focus of this study to match behavioral and model sensitivity.

### The Gated-integrator Model did not Explain the Time Course of Choice Probability

To further investigate the mechanisms of task switching, we examined the time course of the trial-to-trial covariation in MT responses and behavioral choice (i.e. CP). In our model, incongruent MT neurons provided inputs as either task-relevant or task-irrelevant information. Thus, the CP time course of incongruent neurons in the irrelevant task may illustrate the process of switching off the task-irrelevant inputs.

**Figure 4:**
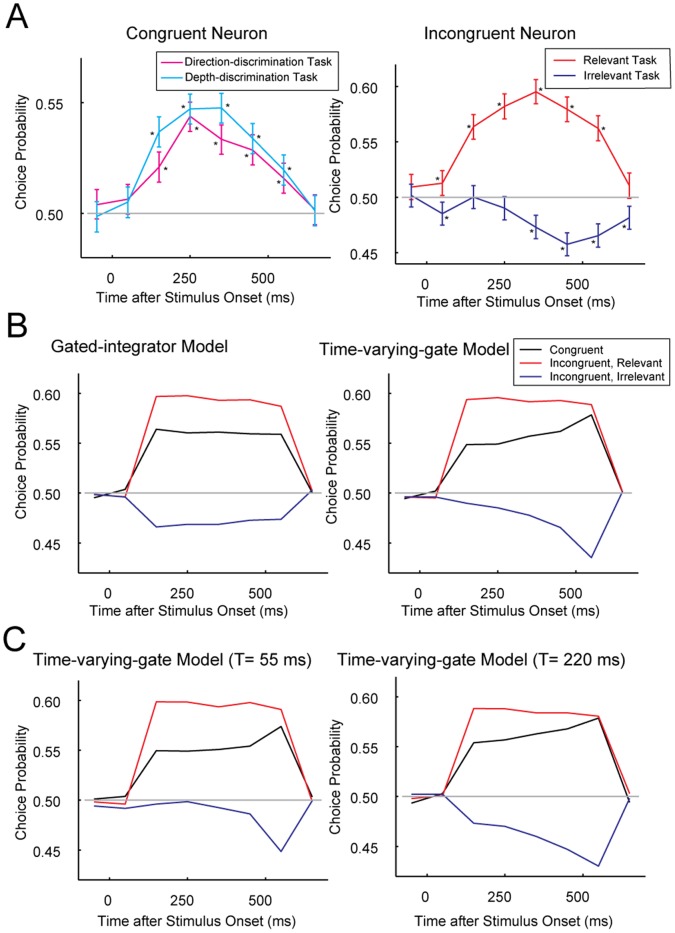
Time course of choice probability (CP). (A) Time course of average CP for middle temporal (MT) neurons. (Left) CP was calculated from 58 congruent neurons separately for the direction- and depth-discrimination tasks. (Right) CP was calculated from 35 incongruent neurons separately for the relevant and irrelevant tasks. Each point indicates CP plotted at the center of a 100-ms time bin. Error bars indicate 95% confidence intervals. Asterisks indicate CPs that were significantly different from 0.5 (bootstrap, *p*<0.05). (B) CP time course for simulated neurons in the gated-integrator model and the time-varying-gate model. (C) CP time course with different time constants.

Sasaki and Uka [5] showed that the CP of incongruent MT neurons initially remained at approximately 0.5 and then gradually dropped below 0.5 near the end of visual stimulation during the irrelevant task (see Fig. 7D in [5]). Here, we recalculated the time course of CP using larger time bins and confirmed that the time course of CP of incongruent MT neurons produced a negative trough near the end of visual stimulation (Fig. 4A). We next computed the time course of CP of the model to test whether the model explained the negative CP trough. The CP of the gated-integrator model dropped below 0.5 from the beginning of visual stimulation and reached a plateau, without showing a negative trough (Fig. 4B left). Therefore, although the gated-integrator model succeeded in explaining the psychophysical data, alternative models were necessary to explain the CP time course.

### A Time-varying-gate Model Explains CP Time Course

As a model to explain the CP time course, we first examined a time-varying-gate model. In the previous section, we showed that the predicted CP of the gated-integrator model did not change over time because the weight was fixed. If the weight is a function of time, CP can also change over time and thus show a negative trough. For example, it seems likely that the weight on the task-irrelevant information gradually decreases because it may take time to shut the gate. However, this predicts a negative trough of CP at the beginning of stimulus presentation, and not near the end. Thus, the weight on the task-irrelevant information needs to gradually increase during task presentation. One seemingly unnatural thing about this model is that it predicts less interference from the irrelevant feature with shorter decision time, which is inconsistent with current views of task switching [13]. Moreover, as will be shown later, the performance of this model improved with shorter decision time (see next section). Putting aside the feasibility of the model, we first show that this model explains the time course of CP.

To compare the time-varying-gate model with the leaky-integrator model explained below, we assumed that the weight increased exponentially to 1 at the end of stimulus presentation. In this case, the only parameter was the time constant of the exponential function and this was determined to match the average behavioral SR of 0.70 (see Fig. 4C in [5]). Figure 3A shows the SR of the time-varying-gate models plotted as a function of the time constant. With a time constant of 110 ms, the SR of the time-varying-gate model matched the behavioral SR of 0.70. Additionally, the CP time course of the task-irrelevant incongruent neurons gradually decreased during stimulus presentation and reached its minimum near the end (Fig. 4B, right). Therefore, this time-varying-gate model explains the negative trough near the end of stimulus presentation. We only examined an exponentially opening gate, but the decreasing trend of CP was not dependent on the exact shape of the weight function. Other gradually increasing weight, e.g. exponential functions with different time constants, also gave similar results (Fig. 4C).

### A Leaky-integrator Model also Explains CP Time Course

Another possibility that can explain the negative trough near the end is leakiness of integrators, as accumulation of information is subject to leakage or decay [23]. If the integrator is leaky, information accumulated early is subject to leakage and does not influence choices. On the other hand, information accumulated near the end does not have enough time for leakage and thus influences choices. Therefore, this model is equivalent to the time-varying-gate model in terms of how information is used when a decision is made, and might explain the negative CP trough near the end of stimulus presentation.

#### Single-leaky-integrator model

**Figure 5:**
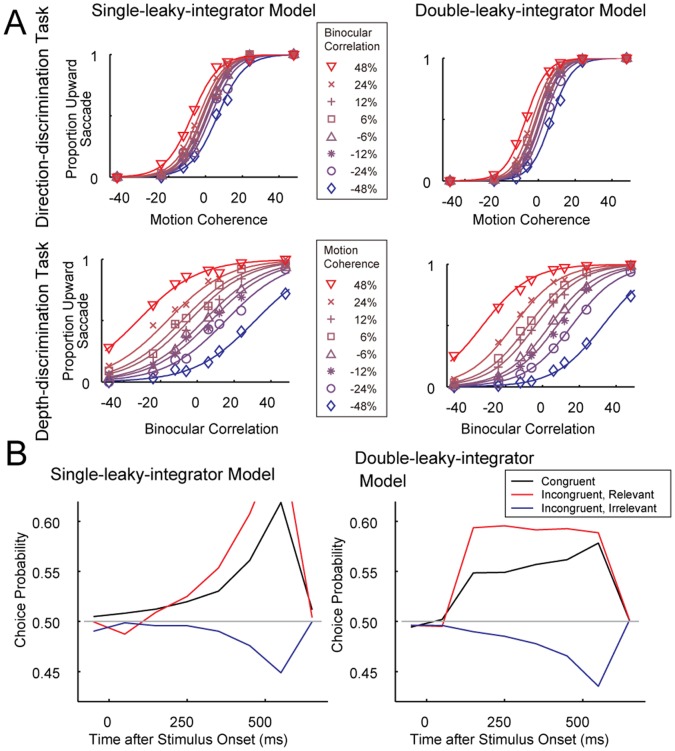
Single-leaky-integrator model (left) and double-leaky-integrator model (right). (**A**) Neurometric functions. (**B**) Choice probability time course.

First we examined a single-leaky-integrator model, which assumed that the integrator was leaky. The leaky time constant was determined to be the same as the time-varying-gate model because we have already shown this to be sufficient to delay the negative CP trough. As the weight on task-irrelevant information, we used the same value as the gated-integrator model. With these values, this model succeeded in explaining the observed behavioral bias (Fig. 5A left), but could not explain the CP time course (Fig. 5B left). The negative CP trough in the irrelevant task was delayed as observed in the time-varying-gate model, but the positive CP peak in the relevant task was also delayed in the same manner. Thus, the effect of leakage was not specific to task-irrelevant information. To explain the negative trough of CP of incongruent neurons, the single-leaky-integrator model is insufficient because it leaks both task-relevant and irrelevant information at the same rate.

#### Double-leaky-integrator model

Next, we assumed that task-relevant and task-irrelevant information were stored separately in two independent integrators. The task-switching process may actively increase leakage from the task-irrelevant integrator while keeping the leakage from the task-relevant integrator negligible. The leaky time constant of the task-irrelevant integrator was determined to be the same as that of the time-varying-gate model. With this parameter assumption, the model predicted exactly the same choices as the time-varying-gate model from the same simulated spike trains, and thus the model SR matched the average behavioral SR. In this leaky-integrator model, task-irrelevant information was subject to leakage and only partially influenced the choice, which explained the behavioral bias (Fig. 5A right). In addition, such leakage affected the task-irrelevant information accumulated early so that CP became close to 0.5 near the beginning of stimulus presentation, but did not affect task-irrelevant information accumulated late so that CP was lower than 0.5 near the end of stimulus presentation (Fig. 5B, right). Thus, this model succeeded in explaining the behavioral bias and the negative trough of choice probability.

### The Leaky-integrator Model was Less Sensitive to Task Commitment Time than Other Models

In the previous section, we introduced the gated-integrator model to explain the behavioral choices and further extended it to the time-varying gate model and the double-leaky-integrator model to explain the negative CP trough near the end of stimulus presentation. Among these models, a characteristic of the leaky-integrator model is that information once stored can be discarded by making the integrator leaky. This might be advantageous when there is little time to prepare for task-switching. As shown in Fig. 2 of Sasaki and Uka [5], the monkeys showed more interference when the tasks were randomly interleaved from trial to trial than when the task was fixed in a block of trials. As a mechanism of increased interference, we inferred that it took time to determine the task during the interleaved trials. If this took longer than the preparation time (300 ms) in some trials, the monkeys may start integration without committing to a task. In such cases, both integrators should initially be either open or closed, and the task-irrelevant integrator becomes either leaky or gated after the task is correctly perceived during stimulus presentation.

**Figure 6:**
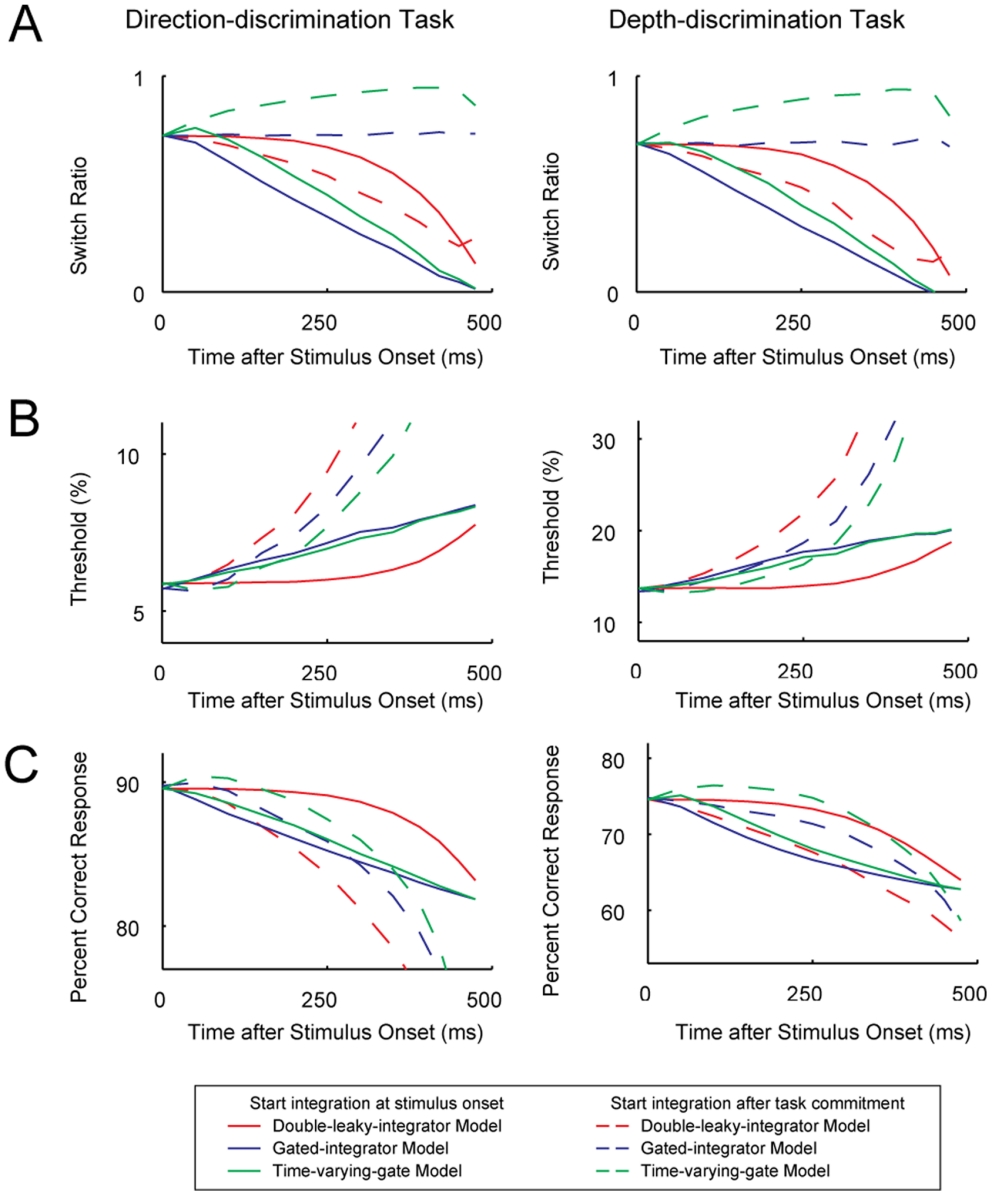
Simulated behavioral performance during late task commitment. Switch ratio (A), 75% thresholds (B), and percent correct responses (C) of the double-leaky-integrator model (red line), the gated-integrator model (blue line) and the time-varying-gate model (green line) with both integrators initially open (solid lines) or with both integrators initially closed (dashed line) were plotted against time when task commitment was completed.

Figure 6 shows the SR (Fig. 6A), threshold (Fig. 6B), and overall percentage of correct responses (Fig. 6C) of the three models as a function of when task commitment was completed. The threshold (75%) shows the sensitivity to the relevant stimulus. In these simulations, model parameters were identical to those used in the previous sections. Therefore, when commitment to a task was completed before integration, the SRs of the models matched the behavioral SR, 0.70.

In the time-varying-gate model with integrators initially open, the overall percentage of correct responses first increased as task commitment was delayed, but quickly decreased with delayed task commitment (green soled line). In the time-varying-gate model with integration starting after task commitment, the effect of the irrelevant information became smaller and SR increased with delayed task commitment because the weight became small soon after integration started (green dashed lines in Fig. 6A). This compensated for the larger threshold (green dashed lines in Fig. 6B) and thus this model showed better overall performance (green dashed lines in Fig. 6C) compared to other models (red and blue dashed lines in Fig. 6C). This means that, under this model, animals could perform better if they just ignored the initial portion of the stimulus and started integration later. If the animals learned to perform the task in this way, the CP of all neurons should be close to 0.5 during the initial part of stimulus presentation. However, Fig. 4A shows that for congruent neurons, CP was significantly different from 0.5 starting 100 ms after the stimulus onset, when stimulus-induced responses arose in MT neurons, which is inconsistent with the time-varying-gate model.

To maximize task-relevant information and minimize the influence of task-irrelevant information, it is best to initially keep both integrators open and then discard stored information from the task-irrelevant integrator (leaky-integrator model: red solid lines in Fig. 6). Leaking stored information from the task-irrelevant integrator after task commitment is beneficial for reducing interference from the task-irrelevant stimuli. In the gated-integrator model, if integrators are initially open (blue solid lines in Fig. 6), stored information in the task-irrelevant integrator is retained until the end, and behavioral choices are easily biased depending on the irrelevant feature of the stimuli, leading to a decrease in SR. To avoid reduction in SR, inputs to both integrators can initially be shut off before task commitment. However, if the integrators are initially closed (blue dashed lines in Fig. 6), sensitivity to the task-relevant feature deteriorates because time for evidence accumulation decreases. Overall, the gated-integrator models soon became inaccurate as it took longer to commit to a task, whereas the accuracy of the leaky-integrator model remained comparable for the first few hundred miliseconds (red solid lines in Fig. 6C). Thus, the performance of the leaky-integrator model did not deteriorate as much as that of the gated-integrator models in cases where the monkeys initially started without task commitment.

## Discussion

In this study, we compared a gated-integrator model, a time-varying-gate model, and a double-leaky-integrator model of task switching by simulation based on the responses of MT neurons. The simulation showed that all models could explain the psychometric functions of the monkeys’ behavior. However, the negative CP trough near the end of stimulus presentation was not explained by the gated-integrator model. The CP time course could be reconciled using the time-varying-gate model, assuming that the input weight gradually increased during stimulus presentation. However, if this gate-opening proceeded simply with time, it predicted less interference from the irrelevant feature with shorter decision time, inconsistent with current views of task switching [13]. We further tested the effect of leakage: without a time-varying gate, the CP time course was reproduced only when the leaky time constants were different between the task-relevant and task-irrelevant integrators (double-leaky-integrator model). Finally, we showed that the leaky-integrator model had better performance than the time-varying-gate model when task-switching took time and animals started integration without committing to a task. Assuming that animals employed optimal computational mechanisms, these results support the leaky-integrator model.

### Model Validity

The models did not fit the monkeys’ data in a variety of situations. First, the simulated sensitivities were different between the two tasks, although behavioral sensitivities were similar (see Fig. 1B in [5]). Additionally, the discrepancy between simulated and psychophysical thresholds appears to deviate from previous studies, which reported similar psychophysical and neuronal thresholds during direction-discrimination [6] and depth-discrimination [10] tasks. Neuronal thresholds of these studies were calculated assuming two neurons: the recorded neuron and a hypothetical antineuron with opposite preference. In our model, six simulated neurons were connected to the task-relevant integrator. The larger number of neurons may explain the higher sensitivity of the model during the direction-discrimination task. In contrast, the simulated sensitivities were lower than the psychophysical sensitivities during the depth-discrimination task. In our task, depth stimuli were not always presented in the preferred directions of the recorded neurons, crucially different from previous studies [10]. Thus, in our simulation, responses of simulated neurons with opposite direction tunings were averaged. Because most MT neurons have stronger tuning to direction than to depth [26] and do not respond well at the null direction, this may have affected simulated sensitivities particularly during the depth-discrimination task. We were able to match behavioral and model thresholds by assuming that neurons with strong depth tuning are preferentially selected for further readout. Specifically, by estimating average firing rates based on a group of neurons with similar tuning strength in both dimensions, the threshold predicted from the model matched behavioral thresholds (Fig. 3C). Although we performed this analysis using the gated-integrator model, the same results were accomplished using the leaky integrator model. Alternatively, combining more inputs for depth discrimination can also decrease threshold. This can be achieved by assuming that more incongruent neurons in the model are connected to the depth integrator. Lastly, noise correlation between neurons is also important to read out information from a neural population [27]. Because our model neuron represents an independent signal from a group of neurons, decreasing noise correlation is equivalent to increasing the number of model neurons. A combination of any of these mechanisms may reconcile the discrepancy between neuronal and behavioral thresholds.

### Choice Probability

CP represents covariation between neural responses and behavioral choices. A high CP does not necessarily indicate a causal relationship between neural responses and behavior, particularly when decisions are based on a large population of neurons [27]. Rather, correlation of noise among neurons in a sensory pool is the deciding factor that governs CP magnitude [27], [28]. In our model, each of the eight model neurons represents a collective activity of a neuron group, and we assumed no noise correlation among model neurons for simplicity. Thus, the weight of inputs to the integrators had a more direct effect on CP magnitude. Because we used Poisson spike generators, we implicitly assumed no temporal correlation for the firing rates. This was critical in our analysis of the CP time course. Implementation of a temporal correlation would greatly affect our results. Future investigations of the temporal firing correlations in MT neurons are of particular importance to validate our model.

Until now, we assumed that CP reflected a feed-forward relationship between neural activity and behavioral choice. However, CP in a later period may have arisen from feedback signals depending on behavioral choice; feedback signals could affect neural responses more prominently later in a trial [29]. More specifically, Nienborg and Cumming argued that the negative CP trough in Sasaki and Uka [5] is exactly what is expected from a negative feedback signal [28]. Indeed, a big feed-forward CP component may mask a feedback component during the relevant task, resulting in a peak CP at the middle of stimulus presentation. In contrast, a CP feedback component may become prominent later during stimulus presentation in the absence of feed-forward components for incongruent neurons during the irrelevant task. In this case, the simple gated-integrator model does not contradict with the observed CP time course.

### Leaky Integrator as a Mechanism for Flexible and Fast Decisions

Sensory input is integrated over time when decisions are based on weak stimuli [12]. Usher and McClelland stated that such integration is subject to leak [23]. We further extended this hypothesis and proposed the novel idea that the amount of leakage may be actively modulated to accomplish task switching. Here we discuss possible mechanisms to modulate leakage assuming that the integrators are implemented by independent recurrent circuits.

Here we consider two ways to alter leakage of an integrator based on the model proposed by Usher and McClelland [23]. These mechanisms may also be applied to a more biologically realistic model proposed by Wang based on spiking neurons [24], which is known to approximate the Usher and McClelland model [23] for certain parameters [30]. The integrators in the above models are composed of neural networks with recurrent excitation [23], [24], [31], and information is stored as the difference between two integrators which are mutually inhibited (lateral inhibition). Because excitatory neurons do not directly inhibit neurons, lateral inhibition is mediated by an inhibitory neuron pool [24]. In spite of the large intrinsic leakage of each integrator, determined by the properties of single neurons, the activity of an integrator is sustained by recurrent excitation, and the difference between the integrators is amplified by lateral inhibition [23]. If the strength of recurrent excitation and lateral inhibition are balanced with the intrinsic leakage of the integrators, information can be stored without leak, simplifying the model to the diffusion model [21]. Taking into account that a trial-by-trial control of the intrinsic leakage and/or synaptic weight is unrealistic, we consider two ways to modulate the effective leakage of the diffusion process. The first is to vary the strength of lateral inhibition. This can be realized by exciting or inhibiting the inhibitory neuron pool. The second is to modulate the strength of recurrent excitation. This can be achieved by varying background input because neurons’ responsiveness to excitatory input is influenced by noisy background input [32], [33]. Our hypothesized mechanisms do not rely on changing single neuron properties such as the time constant of NMDA receptors and/or synaptic weight, but on changing the properties of recurrent networks by activation or inactivation of a subset of neurons according to task. Although further quantitative examinations are necessary, these comprise potential implementations of the leaky-integrator model for flexible and fast decisions.

Our leaky integrator model is implemented in a purely feedforward architecture and requires one integrator for each task. In the case where monkeys are required to perform more than two tasks, the required number of neurons to perform each task should grow linearly. It is possible that different architectures, such as a network where sensory neurons are recurrently connected [34], require less number of neurons. It is of further interest to build and test a recurrent network model that involves both task switching and integration of sensory information relevant to multiple tasks. Parameter modulation to realize leakage may also be easier to address in this type of architecture.

Recently, many studies have shown that activity in the lateral intraparietal area (LIP) represents sensory-input integration in the direction discrimination task [22], [35]–[37]. Our model suggests that separate integrators for direction and depth exist and that the way in which integration commences is modulated by the task. Specifically, a strict implementation of the leaky-integrator model predicts that the irrelevant integrator initially integrates but fails to continue integrating because of the leak, leading to a plateau in responses. Future studies concerning the LIP neuron responses during our task should elucidate whether these predictions can be verified.
